# The Combination of *Tamarindus indica* and Coenzyme Q10 can be a Potential Therapy Preference to Attenuate Cadmium-Induced Hepatorenal Injury

**DOI:** 10.3389/fphar.2022.954030

**Published:** 2022-08-08

**Authors:** Amany Abdelnaby, Nabila Abdel-Aleem, Ayman Mansour, Afaf Abdelkader, Amany N. Ibrahim, Safwa M. Sorour, Enas Elgendy, Heba Bayoumi, Shaymaa M. Abdelrahman, Samah F. Ibrahim, Ilhaam Alsaati, Ahmed Abdeen

**Affiliations:** ^1^ Department of Biotechnology, Animal Health Research Institute, Agricultural Research Center, Giza, Egypt; ^2^ Department of Forensic Medicine and Toxicology, Faculty of Veterinary Medicine, Benha University, Toukh, Egypt; ^3^ Department of Forensic Medicine and Clinical Toxicology, Faculty of Medicine, Benha University, Benha, Egypt; ^4^ Center of Excellence in Screening of Environmental Contaminants (CESEC), Benha University, Toukh, Egypt; ^5^ Department of Pharmacology, Faculty of Medicine, Benha University, Benha, Egypt; ^6^ Histology and Cell Biology Department, Faculty of Medicine, Benha University, Benha, Egypt; ^7^ Medical Biochemistry and Molecular Biology Department, Faculty of Medicine, Benha University, Benha, Egypt; ^8^ Department of Clinical Sciences, College of Medicine, Princess Nourah bint Abdulrahman University, Riyadh, Saudi Arabia; ^9^ Department of Basic Sciences, College of Medicine, Princess Nourah bint Abdulrahman University, Riyadh, Saudi Arabia; ^10^ Department of Biochemistry, Faculty of Veterinary Medicine, South Valley University, Qena, Egypt

**Keywords:** cadmium, *Tamarindus indica*, coenzyme Q10, antioxidants, hepatorenal damage, oxidative stress

## Abstract

Cadmium (Cd) is a hazardous environmental pollutant that menaces human and animal health and induces serious adverse effects in various organs, particularly the liver and kidneys. Thus, the current study was designed to look into the possible mechanisms behind the ameliorative activities of *Tamarindus indica* (TM) and coenzyme Q10 (CoQ) combined therapy toward Cd-inflicted tissue injury. Male Wistar rats were categorized into seven groups: Control (received saline only); TM (50 mg/kg); CoQ (40 mg/kg); Cd (2 mg/kg); (Cd + TM); (Cd + CoQ); and (Cd + TM + CoQ). All the treatments were employed once daily *via* oral gavage for 28 consecutive days. The results revealed that Cd exposure considerably induced liver and kidney damage, evidenced by enhancement of liver and kidney function tests. In addition, Cd intoxication could provoke oxidative stress evidenced by markedly decreased glutathione (GSH) content and catalase (CAT) activity alongside a substantial increase in malondialdehyde (MDA) concentrations in the hepatic and renal tissues. Besides, disrupted protein and lipid metabolism were noticed. Unambiguously, TM or CoQ supplementation alleviated Cd-induced hepatorenal damage, which is most likely attributed to their antioxidant and anti-inflammatory contents. Interestingly, when TM and CoQ were given in combination, a better restoration of Cd-induced liver and kidney damage was noticed than was during their individual treatments.

## Introduction

Cadmium (Cd) is one of the most harmful of environmental and industrial pollutants that endangers human and animal health (Balali-Mood et al., 2021). It is ubiquitously existent in the environment, naturally or *via* pollution (van Gerwen et al., 2022). Contaminated water, food, and air (particularly cigarette smoke) are the most inevitable sources of exposure to Cd (Abdeen et al., 2019). Alarmingly, Cd is poorly excreted out from the body and therefore has the ability to accumulate in various tissues, mainly the kidneys and liver (Taghavizadeh Yazdi et al., 2021; Wang et al., 2021).

It is well documented that Cd has a high affinity to sulfhydryl thiol–containing cellular macromolecules, such as antioxidant components and metallothioneins (MTs), a cysteine-rich protein important for the detoxification of Cd (Wang et al., 2021). The Cd-MT complex is generated and accumulates in liver and kidney cells, causing Cd sequestration in the target site and consequently disrupting cellular redox hemostasis. A building body of studies has shown that oxidative damage is deemed to be the principal mechanism of Cd-induced tissue damage (Abdeen et al., 2019; de Anicésio and Monteiro, 2022). Cd promotes mitochondrial deterioration leading to reduction in ATP synthesis and incremented reactive oxygen species (ROS) generation (Mirkov et al., 2021). As a result, mitochondrial dysfunction, peroxidation of membrane lipid (LPO), protein misfolding, DNA oxidation, and ultimately, overture of apoptotic pathways are instigated (Abdeen et al., 2019; Mirkov et al., 2021; Elgazzar et al., 2022).

*Tamarindus indica* (TM) is commonly known as tamarind, which grows naturally in tropical and subtropical regions (Asad et al., 2022). Many studies have been published the benefits of TM as a natural antioxidant and on its ability to scavenge ROS, which are attributed to its high flavonoid and phenolic contents (Maria et al., 2011; Escalona-Arranz et al., 2016; Devi et al., 2020; Kengaiah et al., 2020). Accordingly, an ample number of literature have reported that TM could exert a hepatorenal protection against a variety of environmental toxicants and drugs, such as toxicities caused by fluoride (Ranjan et al., 2009), carbon tetrachloride (Atawodi et al., 2013), ethanol (Ghoneim and Eldahshan, 2012), antitubercular drugs (Amir et al., 2016), and gentamicin (Khader et al., 2019).

Coenzyme Q10 (CoQ) is a natural antioxidant molecule present in all biological membranes of living organisms (Sangsefidi et al., 2020; Abdeen et al., 2020). CoQ is predominantly found in high energy–demanding cells such as the hepatocytes and renal cells, where the mitochondria are plentiful. It is an integral constituent of the mitochondrial electron transport chain and essential in ATP synthesis (Sangsefidi et al., 2020). Additionally, as a part of the intracellular antioxidant system, CoQ has a substantial free radical scavenging capability, which assists in maintaining the mitochondrial membrane potential and in mitigating LPO; thereby, the oxidative stress is quenched (Gutierrez-Mariscal et al., 2020). The antioxidant potency of CoQ has been documented against oxidative harm done to the liver and kidneys triggered by cisplatin (Fatim a et al., 2013), doxorubicin (El-Sheikh et al., 2012), piroxicam (Abdeen et al., 2020), gentamicin (Upaganlawar et al., 2006), and acetaminophen (Fouad and Jresat, 2011).

Considering the antioxidant potential of TM and CoQ on quenching the generated ROS and promoting the cellular antioxidant capacity at the levels of the mitochondria and cytoplasm, we anticipated that supplementing both the agents might mitigate Cd-stimulated oxidative stress and enhance tissue remodeling. Hereby, the current research evaluates their palliative capability on Cd-prompted hepatorenal oxidative stress. Biomarkers of the liver and kidneys, lipid profiles, oxidative state, and histopathological alterations were all investigated.

## Materials and Methods

### Botanical Material and *Tamarindus indica* Extract Preparation

*T. indica* seeds were gained from the local market (Cairo, Egypt). The seeds were removed manually from their coats, then rinsed and dried at 40°C for 24 h in the dark. Next, the seeds were processed into a powder using a lab blender. Twenty-five grams of dried and smashed seeds were placed in a conical flask and steeped for 3 days at room temperature in 500 ml of anhydrous ethanol, stirring constantly. The obtained extract was filtered before being roto-evaporated, after which the extract paste was freeze-dried. Finally, the TM extract was diluted in saline and thoroughly mixed with vortex before use.

### Phytochemical Profiling of *Tamarindus indica* Extract Using Gas Chromatography–Mass Spectrometer

The phytochemical constituents of TM were identified by GC/MS analysis (GC-Trace Ultra–ISQ mass spectrometer, Thermo-Scientific, Austin, United States) as previously described by our group (Abdeen et al., 2021).

### Assessment of Total Phenolic Content and Total Flavonoid Content

The TPC and TFC of TM were calculated by a colorimetric technique in accordance with the Žilić et al. (2012) technique.

### ABTS^+^ and 2,2-Diphenyl-1-Picrylhydrazyl Radical-Scavenging Efficacy

The scavenging capability of the robust free radicals—2,2-diphenyl-1-picrylhydrazyl (DPPH^•^) and 2,20-azino-bis(3-ethylbenzothiazoline-6-sulphonate) (ABTS^+^)—was assessed according to the Hwang and Thi (2014) method.

### Animals and Ethical Endorsement

From the Center of Laboratory Animal, Faculty of Veterinary Medicine at Benha University, Egypt, 42 Wistar Albino rats (130–150 g) were obtained. Two weeks prior to the incipience of the trial, the rats were housed and accustomed to get adjusted to the environmental conditions (25 ± 2°C) and were supplied balanced pellets and water *ad libitum*. All animal experimentation steps were incongruent with the guidelines set by the ethical committee established in the Faculty of Veterinary Medicine, Benha University (approval no: BUFVTM130222).

### Design of the Trial

Subsequent to acclimation, the animals were sorted into seven equable groups (six rats in each group): Control group—the rats were given normal saline as a vehicle; TM group—TM seed extract (50 mg/kg b.wt) was given to the rats (Sundaram et al., 2014); CoQ group—animals received coenzyme Q10 (CoQ; 40 mg/kg b.wt) (Abdeen et al., 2020); Cd group—the rats received cadmium chloride (CdCl_2_; 2 mg/kg b.wt) (Abdeen et al., 2019); Cd + TM group—the rats were given TM and CdCl_2_; Cd + CoQ group—the rats were administered CoQ and CdCl_2_; and Cd + TM + CoQ group—animals were treated with TM, CoQ, and CdCl_2_ at the same abovementioned dosages. TM and CoQ were given 1 h foregoing to CdCl_2_. All of the remedies were given orally once daily for 28 sequent days. CoQ capsules (Coenzyme Q10^®^, 30 mg) were bought from MEPACO-MediFood, Egypt. While, CdCl_2_ was got from the Central Drug House (P) Ltd., New Delhi, India.

### Sampling

At the end of the experiment, the rats were euthanized under isoflurane inhalation anesthesia. Then, blood samples were immediately sampled from the inferior vena cava, centrifuged for 15 min at 2,000*g* for serum separation, and stored at −20°C for biochemical investigations. To dislodge blood clots, liver and kidney tissues were swiftly harvested and permeated in ice-cold saline. After that, each tissue sample was separated into two halves, one of which was kept in a 10% buffered formalin solution for further histological analysis, while the other part was treated as shown later for the oxidative cascade and biomarkers evaluation.

### Serum Biochemical Indices

Serum alanine aminotransferase (ALT), aspartate aminotransferase (AST), alkaline phosphatase (ALP) enzymatic activities and BUN, creatinine, total protein, albumin, globulin, and lipid profile (cholesterol and triglycerides) were estimated using chemical kits bought from Laboratory Bio-diagnostics Co., Cairo, Egypt. All the procedures were executed in conformity with the manufacturer’s guidelines.

### Preparation of Tissue Homogenates and Oxidative Cascade Analysis

Using a sonicator homogenizer, 1 g of each tissue was homogenized in 5 ml of ice-cold buffer (50 Mm potassium phosphate, pH 7.5, 1 Mm EDTA). Aliquots of tissue homogenates were spun at 4,000 rpm in a cooling centrifuge for 20 min, and the supernatant was frozen at −20°C till further assessment of the oxidative parameters. Oxidative cascade was achieved by measuring the catalase (CAT) activity and malondialdehyde (MDA) and reduced-glutathione (GSH) levels utilizing special diagnostic kits procured from Laboratory Bio-diagnostic Co.

### Histopathological Inspection

Following proper fixation (10% buffered formalin for 24 h at 25°C), the liver and kidney tissue specimens were rinsed with running water for 10 min before dehydrating with successive ethanol dilutions. They were cleared up in xylene solution after that and the tissue specimens were embedded in paraffin at 60°C, then cut into 5-µM-thick sections and stained with H&E to examine the histoarchitectural changes. As previously described by our group, an ordinal scoring system was used to grade the histopathological changes in the liver and kidneys (Abdeen et al., 2021). The scores were graded from 0 to 3 for describing normal (score = 0), mild (score = 1), moderate (score = 2), and severe (score = 3) affections.

### Statistical Analyses

Statistical tests were performed using SPSS software (version 21.0; SPSS Inc., Chicago, IL, United States). Comparisons of the various data sets were done using one-way ANOVA followed by the Duncan’s *post hoc* test. The values are displayed as mean ± SD. Statistically significant values were designated at *p* ≤ 0.05. To determine the contribution of all the variables, the “Factoextra” and “FactoMineR” packages were installed in RStudio under R version 4.0.2 to perform the multivariate principal component analysis (PCA). RStudio and MetaboAnalyst software were used for data visualization.

## Results

### Gas Chromatography–Mass Spectrometer Fragmentation Pattern of *Tamarindus indica* Seeds Extract

**FIGURE 1:**
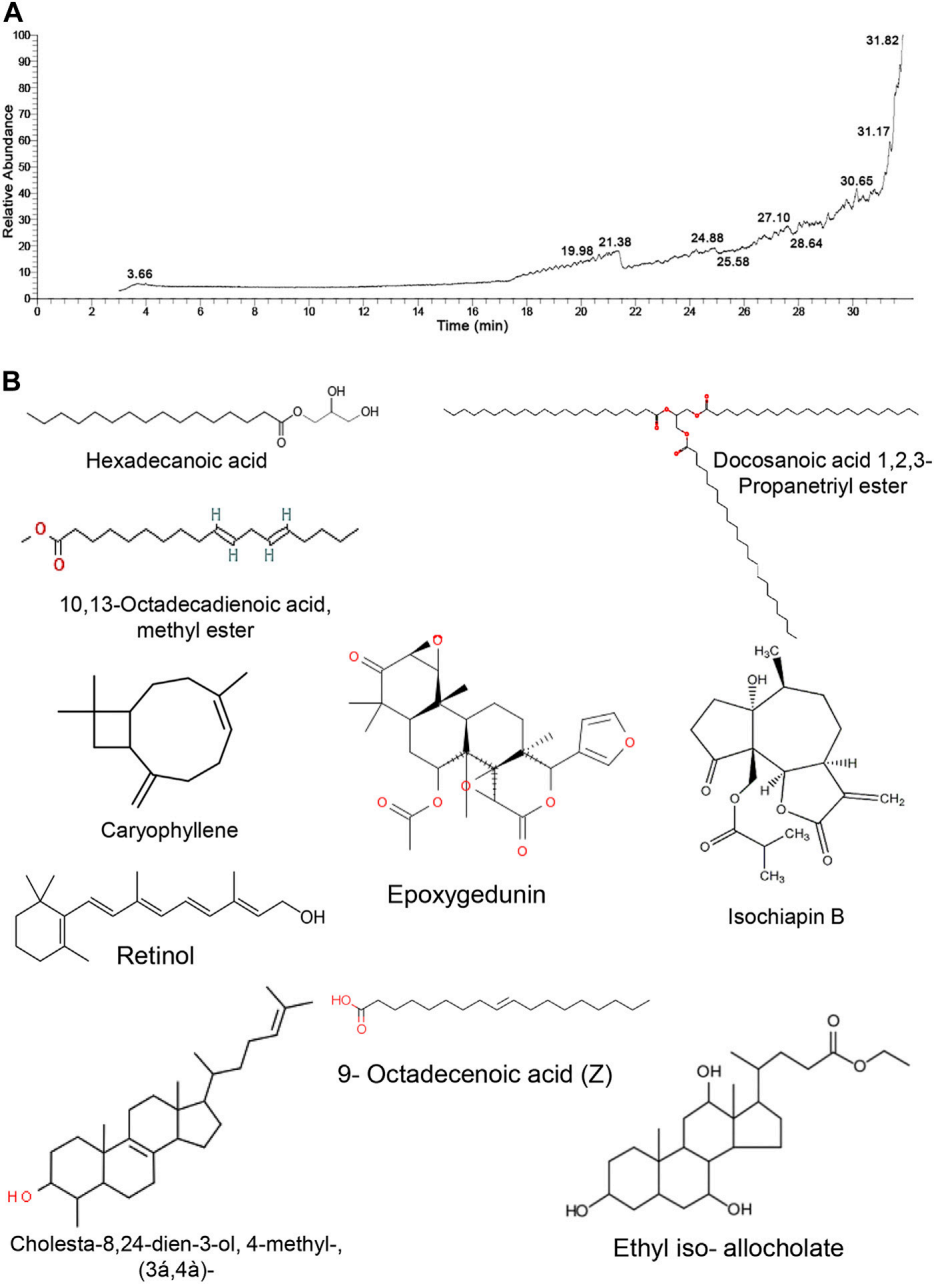
GC/MS analysis of *Tamarindus indica* seeds extract. **(A)** GC/MS chromatogram pattern of TM exhibits the abundance of distinct phytochemical constitutes isolated at various retention times. **(B)** Chemical construction of the identified phytochemicals.

The GC/MS approach was used to identify the chemical components of TM by matching the retention time of the constitutes exemplified in the mass spectra with those obtained from the renowned compounds stored in the spectrometry database libraries. The chromatogram depicted in Figure 1 expounds the mass spectrum pattern of TM, with ten distinct peaks at 3.66, 19.98, 21.38, 24.88, 25.58, 27.10, 28.64, 30.65, 31.17, and 31.82 m/z. The most predominating compounds, which conquered the largest peak area were epoxygedunin (32.24%) followed by caryophyllene (17.91%), ethyl iso-allocholate (11.85%), 9-octadecenoic acid (11.37%), cholesta-8,24-dien-3-ol, 4-methyl-, (3β,4α)- (6.54%), isochiapin B (6.21%), 10,13-octadecadienoic acid, methyl ester (4.63%), tribehenin (3.91%), retinol (3.48%), and hexadecanoic acid, 2,3-dihydroxypropyl ester (1.87%). These specified compounds elucidated that TM is an enriched source of antioxidant and hydrogen donor compounds, explicating its ability to combat many disorders (Table 1).

**TABLE 1:** Phytochemical compounds identified in extract of *Tamarindus indica* seeds by GC-MS analysis.

No	Chemical name	Phytochemical compounds	RT (min)	Peak area %	Chemical formula	MW
1	Hexadecanoic acid, 2,3-dihydroxypropyl ester	Glyceryl palmitate	3.66	1.87	C_19_H_38_O_4_	330
2	10,13-Octadecadienoic acid, methyl ester	Fatty acids methyl esters	19.98	4.63	C_19_H_34_O_2_	294
3	Caryophyllene	Sesquiterpene	21.38	17.91	C_15_H_24_	204
4	Retinol	Vit A	24.88	3.48	C_20_H_30_O	286
5	Cholesta-8,24-dien-3-ol, 4-methyl-, (3β,4α)-	Steroids and derivatives	25.58	6.54	C_28_H_46_O	398
6	Isochiapin B	Terpenoid compounds (sesquiterpene lactone)	27.10	6.21	C_19_H_22_O_6_	346
7	Docosanoic acid 1,2,3-propanetriyl ester (Tribehenin)	Glyceryl tribehenate fatty acid	28.64	3.91	C_69_H_134_O_6_	1,058
8	9-Octadecenoic acid (Z)	Oleic acid	30.65	11.37	C_18_H_34_O_2_	282
9	Ethyl iso-allocholate	Steroid derivatives	31.17	11.85	C_26_H_44_O_5_	436
10	Epoxygedunin	Saponin/steroids	31.82	32.24	C_28_H_34_O_8_	498

MW, molecular weight; RT, retention time.

### Total Phenolic Content, Total Flavonoid Content, and Free Radical Scavenging Potential of *Tamarindus indica*

Table 2 shows that TPC and TFC had average levels of 611.08 mg of gallic acid equivalent (GAE)/g and 179.08 mg catechin equivalent (CE)/g, respectively. Following that, DPPH^•^ and ABTS^+^ were used to evaluate the antioxidant capabilities of the discovered phenolic and flavonoid components. Intriguingly, the gained data elucidated the strong antioxidant activity of TM with average scavenging capacities of 1813.62 and 1703.27 mg TE/g for DPPH^•^ and ABTS^+^ radicals, respectively (Table 2).

**TABLE 2:** Antioxidant contents and activities of ethanolic extract of *Tamarindus indica* seeds.

	ABTS^+^ (mg TE/g)	DPPH^•^ (mg TE/g)	TFC (mg CE/g)	TPC (mg GAE/g)
Replicate1	1711.45	1801.29	179	612.5
Replicate2	1,695.53	1817.14	180	610
Replicate3	1702.81	1822.42	178.25	610.75
Mean ± SE	1703.27 ± 4.61	1813.62 ± 6.36	179.08 ± 0.51	611.08 ± 0.74

TE; trolox equivalent; CE, catechin equivalent; GAE, gallic acid equivalent. Data are expressed as the mean ± SE.

### Serum Biochemical Assays

**FIGURE 2:**
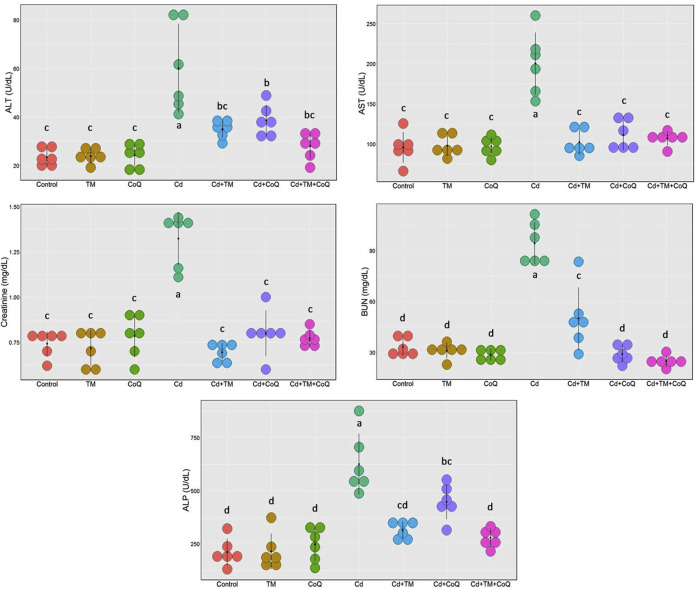
Dot plot of liver and kidney biochemical parameters following treatment with Cd, TM, and CoQ. Values exhibited are mean ± SD (*n* = 6).

**FIGURE 3:**
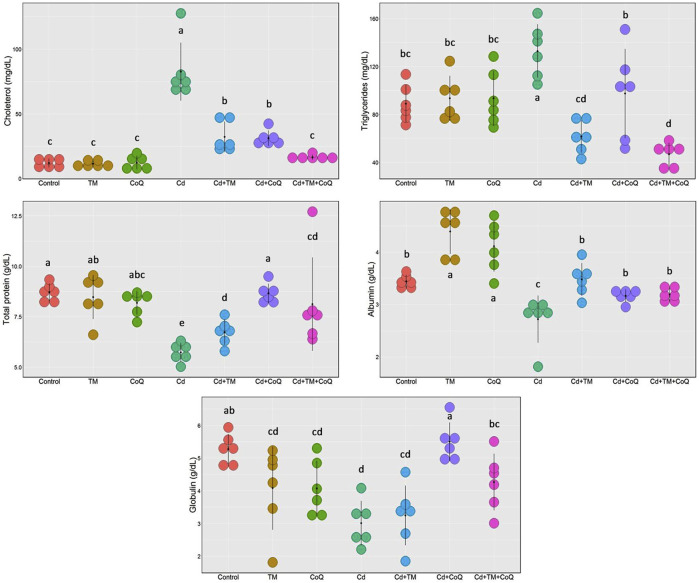
Dot plot of serum proteins and lipid profile following Cd, TM, and CoQ treatment. Cd, cadmium; CoQ, coenzyme Q10; TM, *Tamarindus indica*. Data presented as mean ± SD (*n* = 6).

As shown in Figures 2, 3, Cd treatment resulted in liver and kidney impairment, as documented by marked increases in the serum indices of AST, ALT, ALP, creatinine, and BUN, as well as marked decreases in albumin, total protein, and globulin levels when compared to the Control. We also noticed dramatic increases in cholesterol and triglyceride levels (Figure 3). These findings reveal that Cd poisoning affected lipid metabolism. By contrast, when Cd-exposed rats were given TM or CoQ, the harmful effects of Cd were reduced, as evidenced by a significant improvement in the lipid profile and adjustment of all hepatorenal biomarker levels. As both the medicines (TM and CoQ) were coadministered with Cd, there were notable improvements in hepatic and renal functions and lipid metabolism when compared to their individual treatments.

### Antioxidant and Peroxidation Indices

**FIGURE 4:**
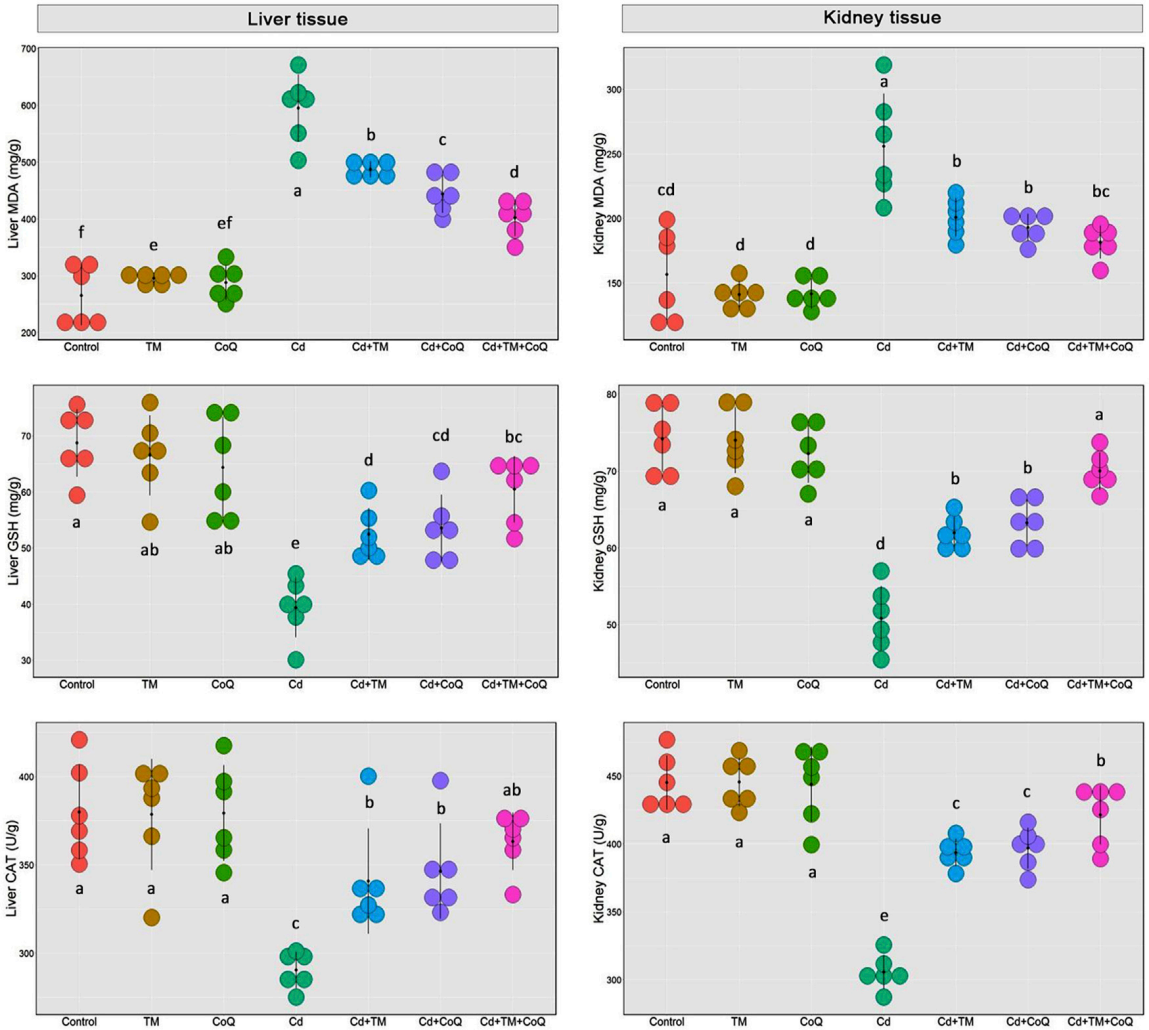
Dot plot of oxidative/antioxidative state following Cd, TM, and CoQ treatment. CAT, catalase; Cd, cadmium; CoQ, coenzyme Q10; GSH, reduced glutathione; MDA, malondialdehyde; TM, *Tamarindus indica*. Data are presented as mean ± SD (*n* = 6).

Lipid peroxidation biomarker (MDA) and antioxidant enzyme capacity (CAT and GSH) upon Cd, TM, or CoQ treatment are illustrated in Figure 4. As explicated, Cd exposure negatively impacted the cellular oxidative state through drastic increment in the MDA levels with an outstanding depletion in CAT activity and GSH level in the liver and renal tissues when compared to the Control. TM or CoQ treatment notably amended hepatorenal oxidative harm prompted by Cd exposure. The overall data revealed a refinement of the oxidative status in the Cd + TM + CoQ group when compared to the Cd + TM or Cd + CoQ group, suggesting that the synchronous use of TM and CoQ has robust synergistic antioxidant advantages against Cd intoxication.

### Histopathological Findings

**FIGURE 5:**
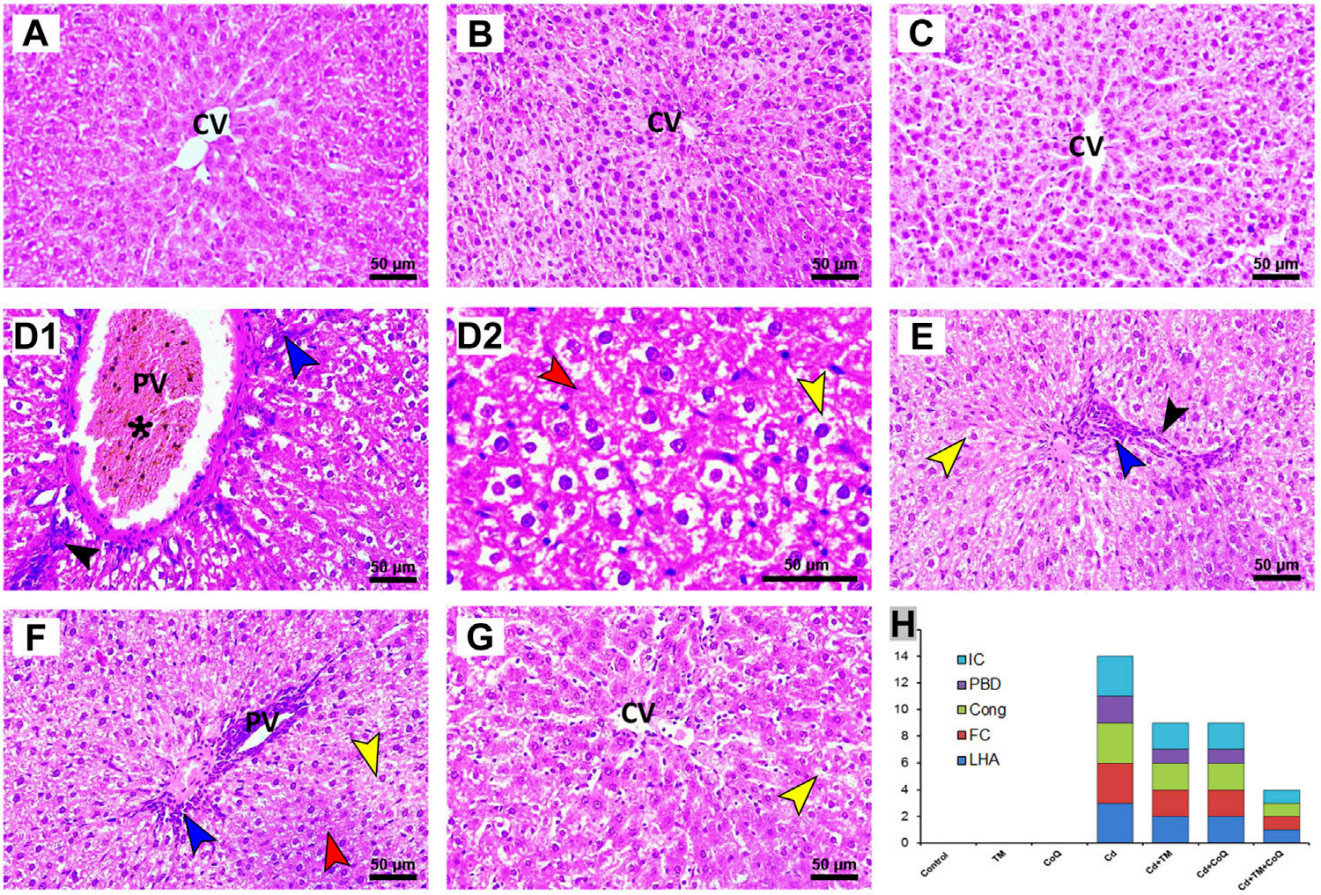
Histopathology of liver tissue in Control and Cd-, TM-, and CoQ-treated groups. Nearly normal hepatic architectures were observed in Control **(A)**, TM-treated **(B)**, and CoQ-treated **(C)** rats. Cd **(D1):** liver section of Cd-treated group showing severe congestion of portal vein (asterisk), proliferation of bile duct (black arrow), and dense portal inflammation (blue arrow). Loss of hepatic architecture (red arrow) and severe fatty changes were also detected in Cd **(D2)**. Cd + TM **(E)** and Cd + CoQ **(F):** dramatic improvement of hepatic architecture observed when Cd was co-administered with TM or CoQ, indicated by mild fatty changes (yellow arrow), portal inflammation (blue arrow), and proliferation of bile duct (black arrow). Cd + TM + CoQ **(G):** rats treated synchronously with Cd and both TM and CoQ revealed hepatic architecture reverted to normal with very mild fatty changes. **(H)** Ordinal scoring of recorded histopathological alterations in the liver tissue. (Cong; congestion; CV, central vein; FC, fatty changes; IC, inflammatory cell infiltration; LHA, loss of hepatic architecture; PBD, proliferation of bile duct; PV, portal vein; scale bar = 50 µM.)

To corroborate the formerly noted observations, the morphological derangement in the hepatic and renal tissues upon Cd, TM, or CoQ administration was evaluated. The hepatic histological inspection of the Control, TM-treated, and CoQ-treated groups exposed normal construction of liver lobule (uniform polyhedral hepatocytes, well-organized sinusoids, and portal veins) as displayed in Figures 5A–C, respectively. On the contrary, the Cd-intoxicated rats showed a loss of hepatic architecture with the proliferation of bile ducts, centrilobular vacuolation of hepatocytes' cytoplasm and diffuse fatty changes “signet cell” (accumulation of fat droplet within the hepatocyte). Besides, portal blood vessel congestion and tremendous leukocyte leakage were also spotted (Figures 5D1,D2). With the concurrent use of Cd, TM, or CoQ, the portal region showed minimal lymphocytic spillage and fatty changes when compared to Cd-untreated rats (Figures 5E,F). Fortunately, when Cd was used synchronously with TM and CoQ, the hepatic architecture mostly reverted to normal with very mild fatty changes (Figures 5G,H).

**FIGURE 6:**
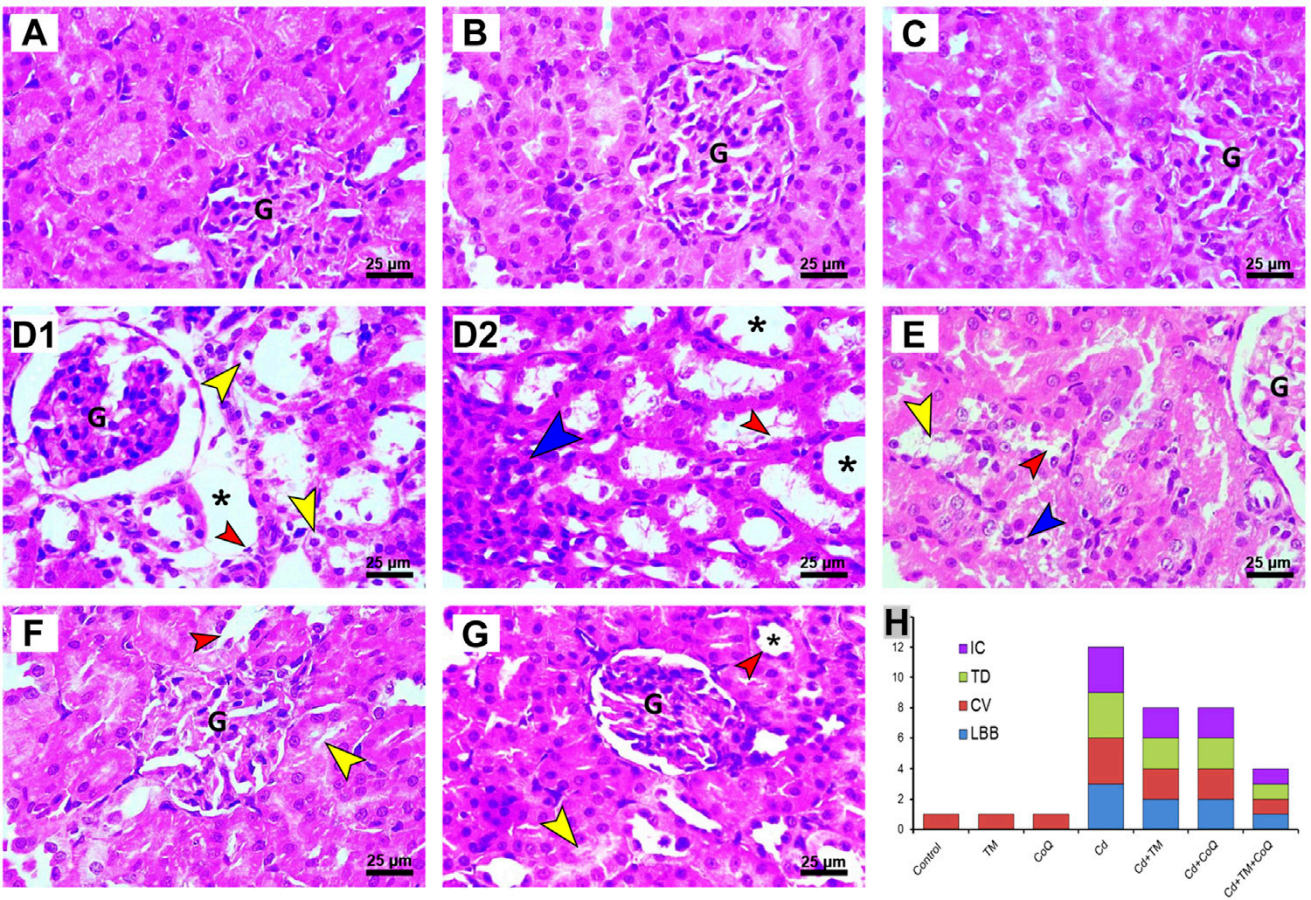
Histopathology of kidney tissue in Control and Cd-, TM-, and CoQ-treated groups. Normal nephrons were observed in Control **(A)**, TM-treated **(B)**, and CoQ-treated **(C)** rats. Cd **(D1** and **D2):** kidney section of Cd-treated group showing loss of normal renal architecture with loss of brush border (red arrow), vacuolated cytoplasm (yellow arrow) in the epithelial lining of renal tubules, dense inflammatory cell infiltration (blue arrow), and severe tubular dilation (asterisk). Cd + TM **(E)** and Cd + CoQ **(F):** dramatic improvement of renal architecture was observed when Cd was co-administered with TM or CoQ, indicated by mild lesions in certain renal tubules. Cd + TM + CoQ **(G):** rats treated synchronously with Cd and both TM and CoQ, expounded that renal tubules were reverted close to normal. **(H)** Ordinal scoring of recorded histopathological alterations in the renal tissue. (CV, cytoplasmic vacuolation; G, glomerular tuft; IC, inflammatory cell infiltration; LBB, loss of brush border; TD, tubular dilatation; H&E, hematoxylin and eosin; scale bar = 25 µM.)

In relation to the kidney sections, the Control, TM-, and CoQ-treated animals (Figures 6A–C, respectively) exhibited no histopathological alterations, with a normal arrangement of the renal parenchyma exhibited by the normal appearance of glomerular tuft, Bowman’s capsule, and tubules. However, the Cd-intoxicated group showed perturbation of renal architecture, desquamation of the apical epithelia of proximal and distal tubules along with cytoplasmic vacuolation. Dense inflammatory cellular infiltration was also noted (Figures 6D1,D2). Nevertheless, combining the treatment of Cd and TM or CoQ culminated in a moderate recovery of histological findings with an improvement of renal architecture as evidenced by mild hydropic changes in the form of vacuolated cytoplasm in some renal tubules (Figures 6E,F). Interestingly, when Cd was used simultaneously with both TM and CoQ, the renal architecture was restored close to normal (Figures 5G,H). The data obtained from the histopathological scoring revealed mitigation of Cd-induced tissue injury in animals co-treated with TM or CoQ, with better improvements when both TM and CoQ were combined. The histopathological findings corroborated the biochemical results, implying that the TM and CoQ supplementation had a considerable impact on Cd-inflicted hepatorenal injury (Figures 5H, 6H).

### Principal Component Analysis and Hierarchical Clustering Heatmap

**FIGURE 7:**
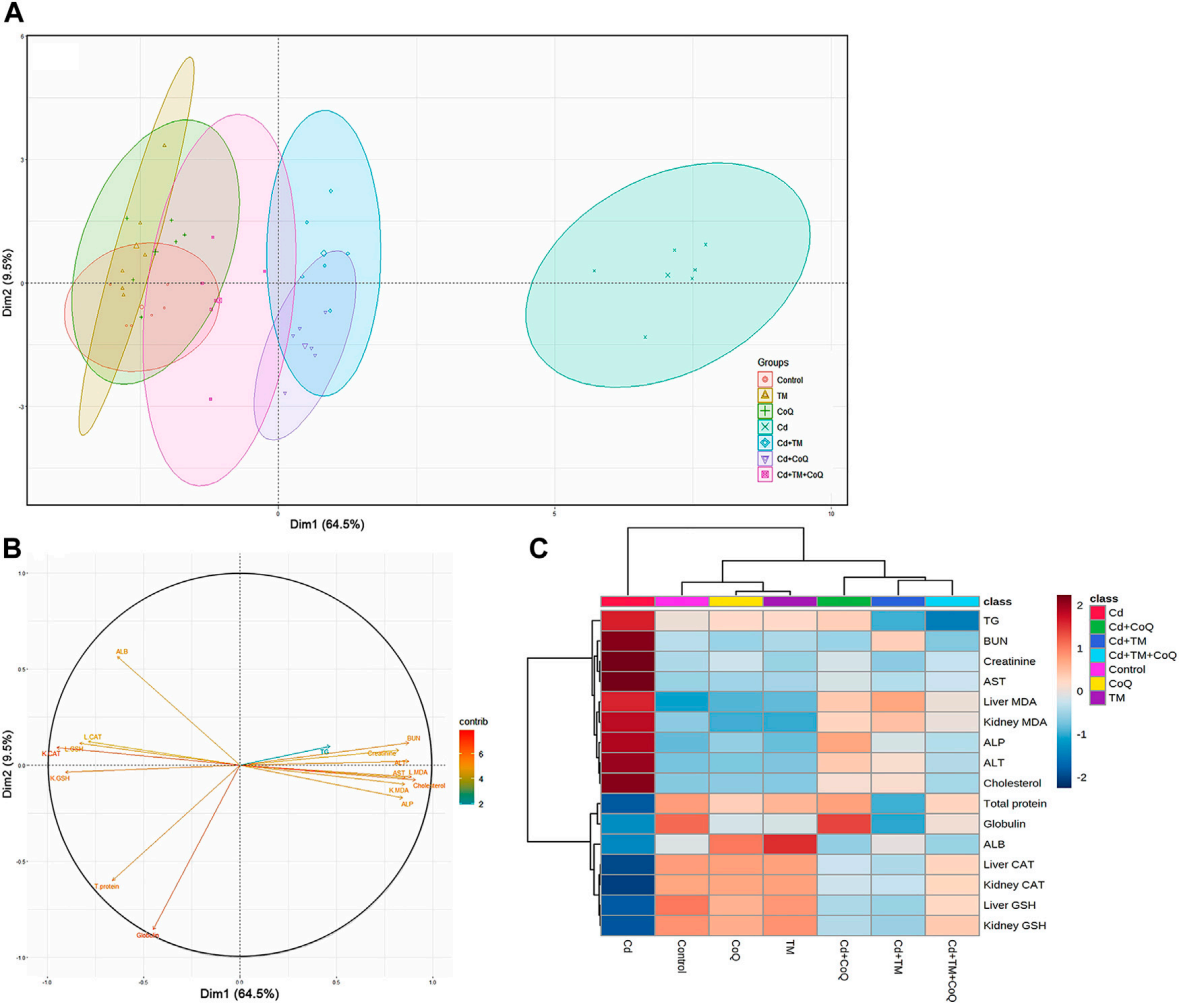
Principal components analysis (PCA) of TM and CoQ against Cd-induced hepatorenal toxicity. **(A)** PCA for discriminating the seven experimental groups (Control, TM, CoQ, Cd, Cd + TM, Cd + CoQ, and Cd + TM + CoQ). Percentage values indicated on the axes represent the contribution rate of Dim1 and Dim2 to the total amount of variations. **(B)** The degree of contribution of various variables. The contribution strength is indicated by a colored scale ranging from the highest (red) to lowest (blue). Cd, cadmium; CoQ, coenzyme Q10; Dim, dimension; TM, *Tamarindus indica*. **(C)** Hierarchical clustering heatmap provides intuitive visualization of all data sets. Each colored cell on the map corresponds to concentration values, with variable averages in rows and different treatments in columns. The gradient scale ranges from dark red (highest value) to blue (lowest value).

All of the studied parameters (creatinine, BUN, ALT, AST, ALP, cholesterol, triglycerides, total protein, albumin, globulin, MDA, GSH, and CAT) contributed mostly along the two major dimensional components (Dim1 and Dim2), accounting for 74% of the total variation. As shown in Figure 7, Dim1 differentiated the majority of investigated factors, resulting in a larger proportion of variance (64.5%), while Dim2 captured the smaller proportion of variation (9.5%). The PCA score plot revealed that the Control, TM, CoQ, Cd + TM, Cd + CoQ, and Cd + TM + CoQ groups were clustered together on the left side and segregated from those subjected to Cd intoxication (Figure 7A). In the PCA loading plot, creatinine, BUN, ALT, AST, ALP, cholesterol, triglycerides, and MDA were positively associated with the Cd group. On the other hand, total protein, albumin, globulin, GSH, and CAT were positively linked with the Control, TM, CoQ, Cd + TM, Cd + CoQ, and Cd + TM + CoQ groups (Figure 7B). Alongside the PCA, the clustering heatmap depicted in Figure 7C provides intuitive visualization of all the data sets, which summarizes the concentration values of all measured biochemical parameters in response to different treatments. These data show how the Cd-treated group exhibited more injury than the other groups.

## Discussion

Cd is a prevalent nonbiodegradable environmental pollutant that threatens human and animal health (Wang et al., 2021). Cd has a long half-life, and once absorbed through the plasma membrane’s calcium channel, it builds up in the body permanently due to its binding to the cytoplasm and nuclear molecules. Cd accumulation in the tissues triggers early oxidative stress and various pathological conditions, particularly in the liver and kidneys (Larregle et al., 2008; Cabral et al., 2021). Oxidative distress is renowned for being initiated when the produced ROS, such as superoxide anions (O_2_^•−^), hydroxyl radicals (OH^•^), and hydrogen peroxide (H_2_O_2_), overrides the endogenous antioxidant's capacity (Abdeen et al., 2019). Thereafter, tissue injury is elicited *via* provoking a cascade of complex mechanisms, such as the enhancement of LPO, protein misfolding, mitochondrial perturbation, depleted ATP production, and DNA damage (Abdel Moneim et al., 2014; Aboubakr et al., 2021a; Wang et al., 2021).

Consequently, the current findings exhibited noteworthy oxidative stress following Cd exposure, as elucidated by significant declines in the GSH concentration and CAT activity in both the liver and kidney tissues. These findings align with formerly published studies (Abdel Moneim et al., 2014; El-boshy et al., 2014; Abdeen et al., 2019). GSH is the major sovereign antioxidant abundantly available in all biological systems since it functions as a nonenzymatic antioxidant by directly scavenging ROS (Abdel-Daim et al., 2021). Cd interacts exclusively with the SH content of the GSH, forming a GSH-Cd complex which is less readily reabsorbed in renal epithelial cells than the Cd-MT complex (Abdeen et al., 2019). The presented findings are in conformity with various former reports proposing that the reduced levels of GSH in the liver and kidneys upon Cd intoxication might enhance their vulnerability to the ROS damaging effect (Renugadevi and Prabu, 2010; Samarghandian et al., 2015; Aboubakr et al., 2021b). In addition to GSH, CAT is another endogenous antioxidant that is necessary for decomposing H_2_O_2_ into H_2_O and O_2_; therefore, it conserves the cell from oxidative harm caused by the oxygen species. Thus, in a condition when CAT activity is depleted by Cd-induced overproduction of ROS, Fenton’s reaction is accelerated and plentiful amounts of OH^•^ are produced (Abdeen et al., 2019). The OH^•^ molecule is the most damaging radical among other ROS, which strongly destroys the lipid membranes, triggering LPO and increasing MDA (LPO marker) levels (Renugadevi and Prabu, 2010).

Alarmingly, besides the damaging effect of ROS, MDA has the competence to make chaotic binding with other subcellular molecules, such as proteins and nucleic acids, speeding up the oxidative cascade, making matters worse (Uddin et al., 2021). Consistently, the current investigation revealed enhanced LPO after Cd exposure, which is indicated by marked increases in MDA tissue levels. It has been reported that when the hepatocyte loses its membrane integrity, the membrane permeability is increased, contributing in the escaping of hepatic enzymes (ALT, AST, and ALP) into the circulation, increasing their blood levels (Abdeen et al., 2021). As shown in the current study, Cd intoxication could dramatically enhance the activities of ALT, AST, and ALP. The histopathological examination vividly mirrored the biochemical findings on the liver sections. Additionally, the activation of the von Kupffer cells and neutrophil recruitment in our study has also been embroiled in the Cd hepatotoxic process. Wang et al. (2021) and Abdeen et al. (2019) have corroborated the hepatotoxic damage in Cd-intoxicated rats.

In other words, histological analysis showed the presence of LPO in renal cell membranes, as seen by the disintegrating apical membrane, which caused tubular dysfunction as exhibited by marked increases in BUN and creatinine levels. There is strong evidence that the proximal tubules’ sensitivity to ROS-induced oxidative damage is due to their high mitochondrial content. Surprisingly, according to the current histological study, the proximal tubules were the most affected areas of the kidneys following Cd intoxication. These findings are consistent with our prior research, which indicates the vulnerability of the cortical region to oxidative degradation caused by different insults such as gentamicin (Abdeen et al., 2021), cefepime (Aboubakr et al., 2021a), and aflatoxins B_1_ (Abdel-Daim et al., 2021). Therefore, it is strongly assumed that mitochondria constitutes a potential subcellular target for Cd (Gobe and Crane, 2010). Furthermore, this work shows substantial reductions in albumin and globulin serum levels which in turn affect the total protein levels after Cd exposure. These reductions might be attributed to the Cd-induced inhibition of protein synthesis due to oxidative stress, endoplasmic reticulum stress, and DNA oxidation, which ended up suppressing mRNA transcription and translation processes in the hepatocytes (El-boshy et al., 2014; Aboubakr et al., 2021a). Our histopathological findings confirm the occurrence of hepatic degenerative changes in response to Cd intoxication. Moreover, the renal reabsorption defects caused by Cd were another condemned mechanism for excessive protein loss in the urine, reducing their serum levels (Abdeen et al., 2020).

Moreover, we observed disruption of lipid metabolism, as seen by raised serum cholesterol and triglyceride levels, implying the presence of liver injury following Cd poisoning, as evidenced by the fatty buildup in hepatocytes shown in our histological findings. Our data are in agreement with those obtained by Larregle et al. (2008), who found that Cd exposure accelerated fat degeneration in isolated rat hepatocytes. It is evident that Cd affects the amount of lipids in various organs. We anticipate that the hypertriglyceridemia and hypercholesterolemia noticed in Cd-treated animals were attributed to the reduced activity of plasma lipoprotein lipase, a pivotal enzyme, in their breakdown (Larregle et al., 2008). Besides, Cd inhibits cholesterol uptake by macrophage, which is crucial in cholesterol metabolism (Ramirez and Gimenez, 2003). Taken together, all the aforementioned mechanisms promote fatty liver induction. These data are in congruence with Kim et al. (2018), who demonstrated a noteworthy rise in cholesterol and triglyceride levels after Cd intoxication in a zebrafish model.

In addition, our results are in harmony with those of Samarghandian et al. Larregle et al. (2008) elucidated that Cd exposure adversely disrupted the lipid profile through LPO. Worse, elevated cholesterol levels were frequently cited as a factor that hastens the deposition of Cd (Türkcan et al., 2015). Additionally, dyslipidemia is reported to promote mitochondrial instability and excess production of ROS, thereby oxidative stress exacerbates the deleterious sequels (Gutierrez-Mariscal et al., 2020).

GC/MS was used to identify the *T. indica* plant in the current study. The GC/MS chromatogram revealed that saponin, terpenoids, steroids, oleic acid, fatty acids methyl esters, vitamin A, and glyceryl palmitate were the most ubiquitous constituents in the TM seed extract (Table 1). Notably, these secondary metabolites evince diverse therapeutic potential, mainly antioxidant (S et al., 2021; Atawodi et al., 2013), anti-inflammatory (Mushtaq et al., 2021), and antihyperlipidemic (Velu et al., 2018) properties. The presence of these phytochemicals is in congruence with that reported in preceding studies those screened the prime constituents of *T. indica* seeds (S et al., 2021; Mushtaq et al., 2021; Sadiq et al., 2016). Furthermore, the current investigation has proven the antioxidant potential of TM by the presence of remarkable levels of phenols (611.08 mg GAE/g), flavonoids (179.08 mg CE/g), and increments of free radicals scavenging activity (according to DPPH^•^ and ABTS^+^ assays), supporting the findings of prior investigations (Rebaya et al., 2015). The phenolic compounds can be deemed the cornerstone of TM’s antioxidant potentiality, depending on their chemical architectures, particularly the number and location of the OH groups, the nature of exchange on the aromatic rings, and double bonds those enable the delocalization of free electrons. They have the potential to transform unpaired electrons to paired ones *via* donating hydrogen atom (H^+^) to them which is crucial for free radicals neutralization and stability, therefore conferring the phenolic molecules their scavenging capability (Yeasmen and Islam, 2015).

Moreover, the OH group has the potential to detoxify the ROS, including mainly HO^•^ radicals aiding in protecting the membrane lipids from the HO^•^-induced LPO. Several reports have revealed a robust correlation between the antioxidant power of plants and phenolic contents (Abdeen et al., 2021). In particular, saponins (epoxygedunin), representing the largest proportion among other constituents of TM (32.24%) in the current study, are well known for their potent antioxidant and chelating activities. Saponins are reported to assist the renovation of mitochondrial function and suppress inflammatory responses (Wang et al., 2021). Likewise, saponins have the capability to scavenge the superoxide radical through the xanthine and xanthine oxidase pathway (Fu et al., 2009).

CoQ is a unique lipid-soluble *de novo*–created antioxidant that confers molecular stability to the membrane phospholipid bilayer (Hernández-Camacho et al., 2018). The most significant and relevant activity of CoQ is associated with the high antioxidant capability of its coexisting redox states (ubiquinone, semi-ubiquinone, and ubiquinol), which function on the electron transport chain (Abdeen et al., 2020). The antioxidant properties of CoQ is located behind its efficiency of inhibiting the electron loss along the electron transport chain, besides its role in the recycling of other antioxidants such as vitamin C and vitamin E (Hernández-Camacho et al., 2018; Gutierrez-Mariscal et al., 2020).

Interestingly, the ongoing experiment suggests that treatment with TM or CoQ could potentially attenuate Cd-induced hepatorenal damage, as seen by remarkable improvements in liver and kidney functions, lipid metabolism, and oxidative/antioxidative state. We also noticed a refinement of histological alterations after TM and CoQ supplementations confirming their ameliorative properties. Our findings are in harmony with antecedent reports that TM could protect rat liver from carbon tetrachloride–induced injury (Atawodi et al., 2013). Another report elucidated the enhancement of antioxidative enzyme activities in the liver and kidneys following CoQ supplement in a piroxicam-intoxicated rat (Abdeen et al., 2020). Besides the antioxidant properties, TM and CoQ have antihyperlipidemic (Velu et al., 2018; Gutierrez-Mariscal et al., 2020) and anti-inflammatory (Abdeen et al., 2020; Mushtaq et al., 2021; da Silveira e Sá Rde et al., 2015) capabilities, which supposedly play a role in restoring serum cholesterol and triglyceride levels and obscuring lymphocytic infiltrates as indicated in our histopathology results, respectively. There are various mechanisms by which CoQ serves lipid metabolism such as boosting fatty acid oxidation and inhibiting the oxidation of low-density lipoprotein, the primary carrier of blood cholesterol, which is implicated in the cumulation of cholesterol (Gutierrez-Mariscal et al., 2020). Intriguingly, saponins in TM cause hypolipidemia by inhibiting HMG-CoA reductase, a crucial enzyme in the cholesterol biosynthetic pathway (Velu et al., 2018). Furthermore, caryophyllene, another essential ingredient of TM, preferentially binds to the cannabinoid type-2 receptor, modulating the inflammatory processes and exerting considerable cannabimimetic anti-inflammatory effects. Caryophyllene can also modulate the release of proinflammatory cytokines (da Silveira e Sá Rde et al., 2015).

We employed multivariate PCA for analyzing the role of TM, CoQ, and Cd interventions on liver and kidney tissues. The PCA displays each experimental group’s pathway in space spanned by biochemical and oxidative stress markers. The data obtained by PCA exhibit how Cd exposure causes a detrimental shift in the measured parameters, rendering these animals differentiated from the other treated groups by about 64.5%. In this work, both single-variate and PCA demonstrated that increasing the antioxidant activity alleviated the detrimental effects of Cd in rats co-treated with TM and/or CoQ, while PCA1 revealed that GSH levels and CAT activities significantly alleviated the damaging effects of Cd. Interestingly, PCA found that administering TM and CoQ together was more effective than using them separately against Cd-induced hepatorenal damage. In addition to the PCA, the clustering heatmap provides intuitive visualization of the concentration values that could potentially discriminate the Cd-exposed group from other treatments.

**FIGURE 8:**
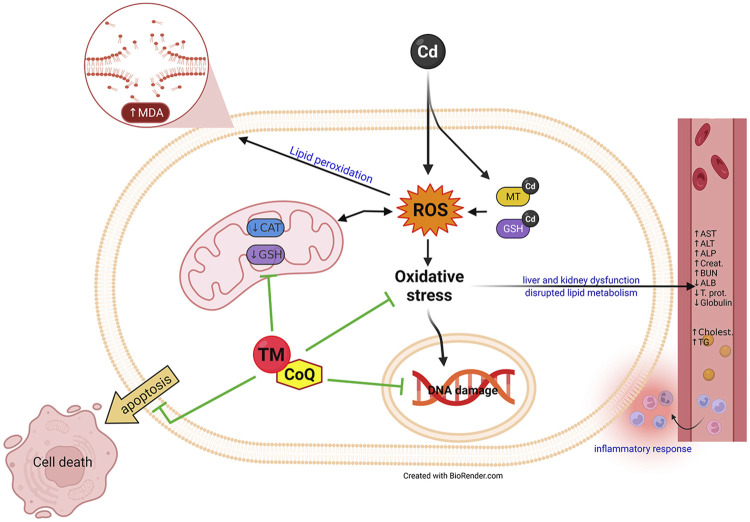
Summary diagram outlines the mechanistic insights underpinning the protective aptitude of TM and CoQ upon Cd-induced hepatorenal damage.

Overall, combining TM and CoQ supplementation provided superior hepatorenal protection against Cd toxicity than did either of the supplements alone. These might be because of their potent ROS-scavenging activity, enhancing the cellular antioxidant system, improving lipid metabolism, and suppressing the inflammatory response. The mechanistic insights underpinning the preemptive potential of TM and CoQ against Cd-induced hepatorenal injury are summarized in Figure 8.

## Conclusion

Cd could evoke notable hepatorenal damage, as demonstrated by higher levels of function biomarkers, changes in lipid and protein metabolism, and changes in oxidative status. TM and CoQ have the capacity to protect hepatocytes and renal cells from Cd-induced damage by combating oxidative distress, which can be because of their antioxidant and anti-inflammatory activities. In Cd-intoxicated animals, supplementing with TM or CoQ only reduces the severity of injury. When both therapies are used together, they produce greater results than when used separately. We expected that supplementing with TM and CoQ would help prevent Cd-induced tissue damage.

## Data Availability

The original contributions presented in the study are included in the article/Supplementary Material, and further inquiries can be directed to the corresponding authors.
